# Planning and proposing evaluations to support project success

**DOI:** 10.1186/s12919-025-00344-2

**Published:** 2025-10-07

**Authors:** Anne E. Leak, Lubella A. Lenaburg, Elizabeth Sciaky, M. Ofelia Aguirre Paden

**Affiliations:** Center for Science and Engineering Partnerships, University of California at Santa Barbara, Santa Barbara, CA 93106-6105 USA

**Keywords:** Evaluation, Data, Proposals, Planning, Goals, Metrics, Program, Broader Impacts, Education Research, Faculty Career

## Abstract

Faculty who are interested in leading grants, education and outreach projects, and other initiatives need evidence to make data-based decisions and demonstrate successes. Evaluations are essential for supporting these efforts and are often required to obtain funding. In fact, a strong evaluation plan within a proposal can ensure alignment between clearly constructed goals and activities in order to promote the likelihood of a project’s success as well as provide reviewers with insight into how the faculty plans to use data in order to make midcourse corrections to ensure success. Once faculty learn how evaluation works and can support the achievement of their research and educational goals, they will be better prepared to successfully plan, implement, and improve projects. In this article, we describe the importance of evaluation, considerations when planning evaluations, and recommendations for working effectively with external evaluators.

## Background

Imagine you are presenting to a National Science Foundation (NSF) site visit team, that asks you questions about how your project is going. You know you have been coordinating activities and mentoring students, but they want to know why some students have not been meeting with their mentor, how many are on track to graduate on time, and whether the students intend to pursue graduate school or a career in your field. Having this data and insight into how your participants are experiencing the program activities allows you to document progress toward the goals you have set.

In another scenario, imagine half of your students have left midway through your program and you do not know why. Not only are you unable to explain this issue to funders, but more importantly you are not sure what to do to address this challenge. An evaluation plan for both tracking students across your program as well as interviewing them each year will provide you with the necessary information to improve participant experiences and increase retention.

The two above scenarios show how evaluation can help you promote successful outcomes as well as support your project to ensure that you use data-based decisions to make improvements. *This paper emphasizes the importance of well-designed evaluations, how to plan and implement them, and tools for working effectively with evaluators across many types of endeavors.*

## Importance of evaluation

Evaluation is a valuable support to Science, Technology, Engineering, and Mathematics (STEM) faculty for research centers, training grants, education and outreach projects, and other initiatives [1]. Effective evaluation can support STEM faculty in several ways, such as by providing data for evidence-based decision making, guiding mid-course corrections to projects, documenting progress toward goals for funders, and tracking participant outcomes over time (e.g., [2]). To minimize bias, it is recommended that the evaluation be carried out by one or more people who are external to the project management, like external evaluators or advisory boards who can provide feedback on progress toward goals [3]. However, there are projects where the project leadership may need to carry out some or all of the evaluation themselves, such as is commonly done with NSF Faculty Early Career Development Program (CAREER) awards [4]. Whatever form it takes, evaluation serves the overall purpose of providing data on the status and progress of a project with respect to identified goals and data-informed recommendations for where to go next.

While many grants require evaluation, its benefits go beyond the fulfillment of a requirement, and it is not uncommon for projects to commission evaluations even when it is not required. *A strong evaluation can provide projects with purposeful data management, a better understanding of what is working or not working in a program, and the ability to spot problems and strengths early and fix/build upon them.*

## Difference between evaluation and education research

Some proposals require faculty to include evaluation and education research components, and in our experience as evaluators and center directors, these two activities are often confused. Evaluation and education research can use similar methods of data collection and analysis, but they differ in the larger purpose and focus [5]. Evaluation connects directly with the project goals and activities and is meant to directly measure the project’s impact and success. Education research, in contrast, is a component of the project and typically a part of one or more of a project’s goals, contributes to a broader knowledge base for teaching and learning, is positioned in existing research, and is disseminated beyond the project leadership and funding organization.

Evaluation can help faculty determine their progress toward and attainment of goals and support ongoing program improvement; therefore, it is essential that evaluation plans directly align with the project goals. Project goals can relate to research products or outcomes, outreach, or professional development to support those learning or teaching STEM.

Evaluation of goals related to the impact or direction of scientific research are often conducted by advisory boards, though research outputs can be tracked by project leaders or external evaluators and provided to advisory board members for their consideration. Advisory boards are typically made up of experts in one or more of the fields related specifically to the project who can guide or advise work at any stage (conception/goal setting, instrument development, data collection, analysis, dissemination) or any part of the project (STEM research, industry connections, professional development). Advisory boards can provide feedback via meetings, e-mail exchanges, phone/zoom calls, site visits, or written reports. Typically, advisory boards are made up of people with a diverse skill set that complement those of the Principal Investigators (PIs) and can fill any gaps in knowledge or skills the PIs may have. While faculty PIs typically have a good understanding of when they would benefit from having an advisory board, a question that faculty often have when writing proposals is when would an external evaluator be important [3, 6].

Evaluators are most often external to project management to avoid bias and maintain impartiality when evaluating progress toward goals or identifying challenges (e.g., [2]). Evaluators can guide and assess work at most or all stages of the project (conception/goal setting, data collection, analysis, dissemination, tracking). They provide feedback via a combination of meetings, e-mail exchanges, phone/zoom calls, site visits, short written summaries, and intensive written reports based on the needs of the project. Written reports can be submitted or integrated into reporting to funding agencies, so it is helpful when evaluators share them as both Word documents and Portable Document Formats (PDFs). Typically, evaluators are individuals or teams with specific skills and experience related to evaluation, statistics/psychometrics, social science research, data management, education research, subject-specific education (e.g., biology education) or a combination of those skills. Evaluators do not necessarily need knowledge that is specific to the goals of the project or its scientific research areas, but should be comfortable talking about the field of the project with participants. Evaluation findings are often only shared internally with project leaders, who then have the option to distribute reports with other team members or funders. Occasionally, evaluators and/or leadership disseminate evaluation findings at conferences or in subject-specific education journals to share project designs, innovations, products, or outcomes more broadly with other STEM educators as was the case for a near-peer mentorship program in chemistry [7]; though such dissemination is more common for education research.

Education research is guided by specific research questions and/or hypotheses and can involve quantitative and/or qualitative methods to help answer those questions, test hypotheses and contribute to a broad knowledge base beyond the scope of the project. In STEM education research, Discipline-Based Science Education Research (DBER) typically focuses on teaching and learning within one field of science (e.g., physics education, biology education, chemistry education, engineering education). What makes DBER stand out from other education research is that it has a “deep grounding in the discipline’s priorities, worldview, knowledge, and practices” [8]. In recent years, DBER has benefitted from alliances across, between, and within specific disciplines (e.g., microbiology, organic chemistry) to support the advancement of STEM education, increase workforce development, broaden participation, and address complex societal challenges [8]. Yet, DBER researchers tend to have roots and do work within a specific science department and can be valuable for furthering goals in developing courses, curriculum, or assessment tools related to the project as part of conducting educational research studies as in this example of research on students’ varied interpretations of arrow use in biology textbooks with implications specific to undergraduate biology teaching [9]. Science education researchers, on the other hand, tend to work within education departments (or even other social science departments such as communication, sociology, etc.) and can be valuable for furthering goals related to science persistence, community-building, or other goals related to the project as part of conducting educational research activities. The field of Science Education Research can focus across science (and even more recently engineering) fields and traditionally emphasizes learning at the pre-college level, but has grown to include higher education as well as research related to curriculum/standards, policy, workforce related research, and other topics that expand knowledge on how to effectively teach and learn science. *Though the methods used for educational research and evaluation are similar, education research explores a specific area of teaching or learning science for broader dissemination while evaluation is essential for using data to ensure that a project is making progress toward its goals.*

## Planning an evaluation

**Fig. 1:**
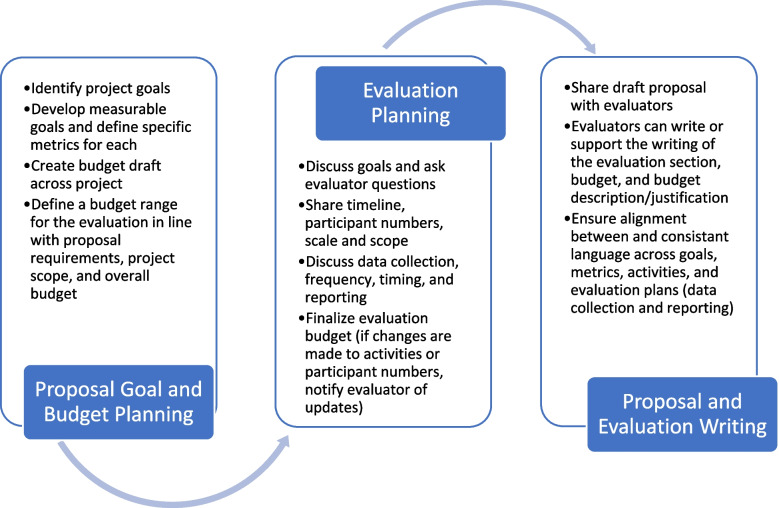
Evaluation planning flowchart showing what faculty should be prepared to think through with their evaluator and information to share with them

The overall process our evaluation team uses in planning an evaluation is first to identify *goals* (targets for guiding and measuring success), *metrics* (specific indicators for monitoring and measuring impact), and *activities* that align across the project and budget range appropriate for evaluating these (e.g., [5]). In many cases, though, the budget dictates the goals, metrics, and activities and an evaluation will need to be appropriately scaled for the funds available. Then, faculty will need to reach out to an evaluator to discuss what data is appropriate to collect, how often to collect data, what type and frequency of reporting is most helpful to provide feedback over the course of the project, all while ensuring that the budget allows for these plans. It is important to start working with an evaluator early in the proposal stage whenever possible (e.g., [2]). The following flowchart shown in Fig. 1 is helpful to consider when developing plans with your evaluator. The planning process will be discussed further in the sections that follow.

## Questions to consider and ask evaluators

When selecting potential evaluators, it is important that the individual or team has a background with data collection, data analysis, and data management. Training and experience can come from social science fields (e.g., education, psychology, communication), statistics or psychometrics, or from STEM education, assessment, and outreach. When meeting with evaluators, faculty should ask what services they offer related to tracking current and former participants, collecting and analyzing data, reporting findings, and data storage and management. Evaluators are not the same as education researchers, but institutional IRB (Internal Review Board) offices vary on whether or not evaluation plans need human subjects review like education research typically does.

One of the most important considerations when talking with evaluators is that the cost of the evaluation needs to align with plans. For instance, the cost to evaluate a project with 5 interns for three years will often be much smaller than the cost to evaluate a center with over 50 student and faculty members over five years. Before having conversations with evaluators, you will need a sense of the project scale (how many participants), scope (what data will be useful to collect from participants), and timing (when does your project start, how long does it run, how long does the evaluator need to continue tracking participants). Most importantly for ensuring this alignment, faculty will need to identify specific goals and metrics and align these with project activities. The questions below will help with proposal planning, evaluation planning, and alignment and should be discussed with the evaluators:

- 1.What activity(ies) are you planning? Why?
- 2.What impact do you want to have? Why is it important? Do you have baseline data to make your case?
- 3.What are your measurable goals and appropriate metrics given existing baseline data?
- 4.What data needs to be collected to know if you are making progress toward your goals?
- 5.How and how often will data be collected to measure your goals?
- 6.How and when will feedback be collected for improving your activity before offering it again?

## Constructing goals and metrics

The most important part of planning any evaluation is developing clear, measurable, feasible goals and metrics. Often, an evaluator can help articulate or focus project goals to ensure that they are feasible within the project’s timeline and budget, but faculty will need to have a sense of what they hope to accomplish in order to plan a well-aligned evaluation for a project. While this may sound simple in theory, it can be a real challenge for project leadership to agree on their goals and metrics and to articulate these clearly. But taking the time to do this early is not only important for the evaluation, but also for developing your activities, programming, and resources.

While goals can take many forms, it is important not to confuse the proposed activities, such as “create a new course for graduate students” or “host 10 community college interns each summer” with the goals needing to be evaluated. The SMART goals acronym was introduced in management and has been mainstreamed as a tool to plan goals for evaluation. SMART goals are Specific, Measurable, Attainable (with appropriate resources), Relevant, and Time-bound. Considering each of these aspects within the context of the project can be useful for planning goals [10]. Overall, goals should reflect the impact faculty want to see as a result of people participating in the project’s activities. Consider the summer internships for community college students, why is this experience being offered? Is it to help recruit students to STEM? To help them be more competitive for transferring to a 4-year university? To encourage them to transfer to your university in an effort to improve recruitment? To prepare them for the STEM workforce? To excite them about graduate school? Not everyone has the same motivation for offering summer internships to community college students, and these motivations not only will shape recruitment, selection, and programming, but also the goals that will help determine if the project succeeded.

Metrics are more specific, data-based indicators of success that can provide a reasonable judgement for whether and to what extent goals are met and can help monitor and measure impact of the project. Metrics may be set in relation to baseline data or to show increasing trends over time. For example, if you know that in your area, only 40% of community college students transfer to STEM bachelor programs, you may decide you would like to see at least 60% of your interns follow this path. Or you may want to see that the percentage of interns in each cohort who identify with a certain group (e.g., from a specific discipline or region) increases over time. Metrics may also help evaluate whether your project is making progress towards a goal even if it is not possible to determine if the goal was met by the end of the award period. For example, if the goal behind offering internships to community college students is that they get into STEM graduate programs, but it’s only a three-year grant, it is not enough time to see whether all interns achieve this goal. However, helpful indicators that show they are on this path could include looking at whether they apply to transfer to a STEM major, if they participated in additional research experiences, whether they apply to STEM graduate programs (including taking the GREs and visiting graduate schools), and whether they earn their STEM bachelor’s degree.

## Determining what data to collect and when to collect data

The project goals, scope, and budget will determine how often data is collected for the evaluation. For most projects, it is recommended that the budget should allow for evaluation data to be collected each year, and often at multiple points during the year, rather than only at the end of the award (e.g., [11, 12]). This not only provides data useful for the annual reporting most funding agencies require, but also allows the leadership to monitor progress throughout the award and identify and address any issues in a timely manner. For evaluating some goals (e.g., recruitment), data collection can occur even before activities begin, through applications or pre-surveys.

Typically, a robust evaluation will use a variety of data types and data collection times to triangulate findings and show likely relationship between program activities and outcomes. While control groups are appropriate for research, they are not always feasible or necessary for evaluation. Project leadership are reluctant to select participants based on random selection, and ethically they do not want to deny opportunities to those who deserve it for the sake of a randomized experiment. Science faculty are also reluctant to confine their evaluation to a fixed structure since their research itself is innovative and often their program, and even goals, need to be able to adapt over time. These reasons are why many evaluators recommend setting goals and metrics with reference to baseline data if comparisons are desired, while maintaining flexibility to revisit these over the course of the program [12]. While you are unable to make causal conclusions that participants were successful because of your program, you can still claim they were successful and show evidence that your project met its goals. You can also document how and why any changes in the program were made from comparisons to initial baseline data. Baseline data may come from your institution, or published studies, or from prior projects. While helpful, baseline data should be carefully selected to closely align with your participants and your program context.

In some cases, funding agencies may require certain data to be reported, either by the project or directly by the participants. For instance, NSF Scholarships in Science, Technology, Engineering, and Mathematics Program (S-STEM) projects include the submission of participant data and updates on academic progress for each participant by term, including their GPA, funds received, and participation in S-STEM activities. NSF NRT projects require participants to complete surveys and upload outputs including publications and presentations. The challenge with funding agencies collecting data directly is that faculty will not have access to de-identified findings or recommendations based on this data. Therefore, it is often necessary for evaluators to collect the same or similar data themselves to ensure that mid-course corrections can be made by the project. For instance, faculty may need to know how well they are meeting recruitment goals or if they should try different recruitment approaches. If only NSF collects this data, the evaluator will still need to collect this information and make recommendations on how to improve recruitment of targeted groups (e.g., students from a specific major). Other times, faculty and evaluators will need to determine what data is most appropriate for measuring progress toward each goal if there is no indication of what is needed for a specific proposal. In this case, faculty should look to institutional or department data as well as existing research to determine what goals are most important for the project and what data would be most helpful in evaluating progress toward these goals.

## Data analysis and reporting

Once the evaluators collect appropriate data for the project, the data should be analyzed and reported to the faculty leadership. Faculty leadership then can integrate evaluation reports into their own reports to funders and/or attach the evaluation reports as separate documents. Reports can be made for each data collection cycle, for instance after a focus group conducted mid-way through an activity. Longer annual reports can be made looking across multiple forms of data to report on progress for each goal along with evidence backing up that progress and relevant recommendations. While many STEM faculty and funding agencies prefer to see numerical summaries of data, it is important to remember limitations in the assumptions of statistical tests and that typically only descriptive statistics are appropriate. This is because projects often have limited sample sizes, but even when they have larger number of participants, there is usually too much bias introduced from how participants join a project for findings to be considered generalizable. Therefore, reports may contain tables and graphs with frequency counts or percentages that can be compared with metrics set for each goal.

Evaluators make use of a variety of analysis methods and tools, most commonly descriptive statistics and qualitative coding. Statistical tools (e.g., Excel, R, SPSS) can be used to analyze quantitative and qualitative data and make appropriate tables and graphs [13]. Qualitative data collected from interviews, survey free-responses, observations, or written documents (e.g., student work, website content, etc.) can be analyzed for frequency counts of specific codes, but more often will be thematically analyzed to reduce and explain findings as they relate to each goal. Text-based analysis software (e.g., Lumivero NVivo, Dedoose, Excel), can be used to code parts of text and find patterns across text. For interviews and focus groups, audio files can also be analyzed directly and themed to find patterns across participant responses or transcribed and coded as text [14, 15]. Analysis methods do not often need to be specified in proposals, but it is important to ask what methods evaluators can use and may be helpful to have a variety of methods used for each project.

One of the reasons it is important to have an external evaluator on projects is to minimize bias in the way data is collected, analyzed, and reported. Evaluators should be honest and balanced with findings and recommendations, so that faculty are aware of problems they should address as well as successes along the way. For reporting, evaluators can also take the role of storyteller to paint a picture of the most significant change from projects, sharing details about the change process with respect to concrete outcomes [16]. When the evaluator is not part of the management structure, they can take a broad and impartial look across projects to figure out progress with respect to goals, how the project got there through stories of change, and what can be done to best reach those goals. It is helpful, especially when doing evaluations internally, to use the analysis of multiple forms of data on an ongoing basis to guide decisions [17]. Evaluation feedback can be used in a transformative way throughout the course of grants, education and outreach projects, and other initiatives to make changes, convince others of necessary decisions, and promote progress toward goals.

## Conclusions

*When planning an evaluation, it is essential to have alignment between goals, metrics, and activities. Working with an evaluator from the proposal stage can be helpful for defining goals as well as considering how data can be collected and analyzed to provide evidence for making decisions and documenting progress toward goals. Evaluation feedback based on this evidence can be shared in a variety of ways, from written reports to virtual meetings. Most important is ensuring that feedback can be used to make midcourse corrections and provide insights into how well goals are being met and if not, why. In this way, evaluations can be planned, proposed, and implemented to support project success.*

## Data Availability

Evaluation data referenced in this manuscript are identifiable and may not be shared openly; however, de-identified aggregated data or data collection instruments may be available from the corresponding author, pending project evaluator and PI approval, upon request.

## Abbreviations

NSF: United States National Science Foundation
STEM: Science, Technology, Engineering, and Mathematics
CAREER: Faculty Early Career Development Program
S-STEM: Scholarships in Science, Technology, Engineering, and Mathematics Program
